# Minimal tillage and intermittent flooding farming systems show a potential reduction in the proliferation of *Anopheles* mosquito larvae in a rice field in Malanville, Northern Benin

**DOI:** 10.1186/s12936-020-03406-2

**Published:** 2020-09-14

**Authors:** Innocent Djègbè, Merdie Zinsou, Edia Flavien Dovonou, Geneviève Tchigossou, Murielle Soglo, Razack Adéoti, Brice Gbaguidi, Seun Atoyebi, Fabrice Chandre, Martin Akogbéto, Jo Lines, Rousseau Djouaka

**Affiliations:** 1 National University of Sciences, Technologies, Engineering and Mathematics, Ecole Normale Supérieure de Natitingou, BP 72, Natitingou, Benin; 2 Laboratoire d’Hydrologie Appliquée, University of Abomey-Calavi, Institut National de l’eau, BP 526, Cotonou, Benin; 3 International Institute of Tropical Agriculture, 08 BP 0932, Cotonou, Benin; 4 UMR IRD 224-CNRS 5290-Université de Montpellier. MIVEGEC. Maladies Infectieuses et Vecteurs : Ecologie, Génétique, Evolution et Contrôle, 911 Avenue Agropolis, BP 64501, 34394 Montpellier Cedex 5, France; 5 Centre de Recherche Entomologique de Cotonou (CREC), 06 BP 2604, Cotonou, Benin; 6 London School of Hygiene and Tropical Medicine, London, UK

**Keywords:** Malaria, Intermittent flooding, Continuous flooding, Minimal tillage, Deep tillage, *Anopheles* larvae, Rice field, Malanville

## Abstract

**Background:**

Irrigation systems have been identified as one of the factors promoting malaria disease around agricultural farms in sub-Saharan Africa. However, if improved water management strategy is adopted during rice cultivation, it may help to reduce malaria cases among human population living around rice fields. This study aimed to assess the impact of the different irrigation practices on malaria transmission, as well as to evaluate the water management system that will best mitigate malaria transmission in Malanville, Benin.

**Methods:**

Knowledge, Attitude and Practice (KAP) study was conducted on 104 households staying on and around the rice fields in Malanville. The study focused on the frequency of mosquito bites and preventive measures against malaria as well as soil preparation and rice planting methods. Mosquito larvae density was assessed in different water management system: continuous flooding (CF) or intermittent flooding (IF), deep tillage (DT) or minimal tillage (MT) and normal levelling (NL) or abnormal levelling (AL) in an experimental hut set-up. Larvae were collected using dipping methods and their density was determined.

**Results:**

Three tillage systems, which include the use of tiller, plow and hoe, were identified on the rice field. Continuous flooding was the only irrigation system used by farmers. Retrospective data from Malanville Health Centre revealed higher malaria cases during rice production season, which was also confirmed by field participants. The density of *Anopheles* larvae was reduced by 80.8%, 30.8% and 40.7% (P = 0.000) during transplanting, tillering and maturation periods, respectively with intermittent flooding compared to continuous flooding. In addition, a clear reduction of larva density was observed with both intermittent flooding systems applied to minimal tillage (MT + IF + NL) and intermittent flooding applied to deep tillage (DT + IF + AL), showing that intermittent flooding could reduce the abundance of malaria vector in rice fields.

**Conclusion:**

Recommending intermittent flooding technology for rice cultivation may not only be useful for water management but could also be an intentional strategy to control mosquitoes vector-borne diseases around rice farms.

## Background

Malaria is a major health problem in Benin, where it is the main cause of morbidity and mortality particularly among children under five and pregnant women [1]. According to the World Health Organization (WHO), an estimated 219 million cases of malaria occurred worldwide resulting into 435,000 deaths [2].

Benin was enlisted among the 10 highest burdened countries in Africa, with a reported increase of malaria cases in 2017 [2]. Although the vast majority of malaria cases occur in rural areas where agricultural activities are high, most of the developmental interventions in these areas are often towards irrigation project and farming improved practices, with very few intervention studies targeting the agro-ecosystem as a contributing factor to the spread of diseases [3].

Inappropriate practices in agriculture, such as poor irrigation systems and continuous flooding of plots, as well as aggressive land use through excessive tillage have been reported to favour the development of *Anopheles* mosquitoes and, therefore, worsen the risk of malaria transmission in rice agro-ecosystems throughout Africa [4–6]. Also, there are reports of higher *Anopheles* mosquito densities in irrigated compared to non-irrigated rice fields [7–9]. The main reason for this assertion is because irrigation systems could, unintentionally, create favourable breeding site for malaria vectors, thereby enabling vector abundance and increasing malaria risks among human populations [6, 10, 11].

Recent studies conducted in rice fields in Mali and Cameroon showed that excessive tillage increases water beds in agricultural settings and extends the duration of standing water pockets therefore allowing malaria vectors to easily complete their developmental cycles [12, 13]. Their reports further revealed that poor handling of these irrigation systems, such as excess watering and continuous flooding of plots, currently performed by farmers during rice cultivation, can lead to increasing breeding sites for mosquitoes and favour the proliferation of malaria vectors [12]. There are other evidences that have associated rice fields with increased *Anopheles gambiae* larvae growth and development [4, 7, 14]. This heliophilic species thrives well in shallow, inundated fields during tilling, transplanting, which runs through the first 6 weeks of rice planting (until canopy closure) and after harvest [15]. Similar events have been recorded in Kenya [16, 17], Burkina Faso [18], Gambia [19], Madagascar [20], Senegal [21] and Mali [22].

However, intermittent flooding system that was suggested for rice cultivation requires the release of water to rice fields when needed [23]. This is not only good for water management but also for reducing mosquito proliferation. This system helps to reduce standing waters in rice plots, which minimizes the abundance of mosquito and consequentially lessen malaria risks among human population [24]. A case study in Kenya has already shown that intermittent flooding has the capacity to effectively control the development of *Anopheles funestus* and *Anopheles coustani* in rice fields. An alternative system, alternate wetting and drying irrigation (AWDI) cycles coupled with constant cleaning of irrigation canals for efficient water flows can also help to reduce the breeding of malaria vectors. There is a case study in Indonesia showing that a cycle of 9 days wet and 2 days dry in 15 ha field trials reduced the density of the malaria vector *Anopheles aconitus* by 75% [24].

Rice is a staple for Africans and its increasing demand means there is need for more production. However, there must be adequate and intentional operations to ensure that increased agricultural activities do not negatively impact other aspects like health and the environment. The negative impact of these operations most times may have effect on the overall social and economic systems.

This study, aimed to investigate common practice in agriculture help in the increased proliferation of malaria vectors specifically, to assess the impact of minimal tillage and intermittent flooding of rice plots on the proliferation of *Anopheles* mosquitoes and malaria risks in rice agro-eco-systems.

## Methods

### Study site

**Fig. 1:**
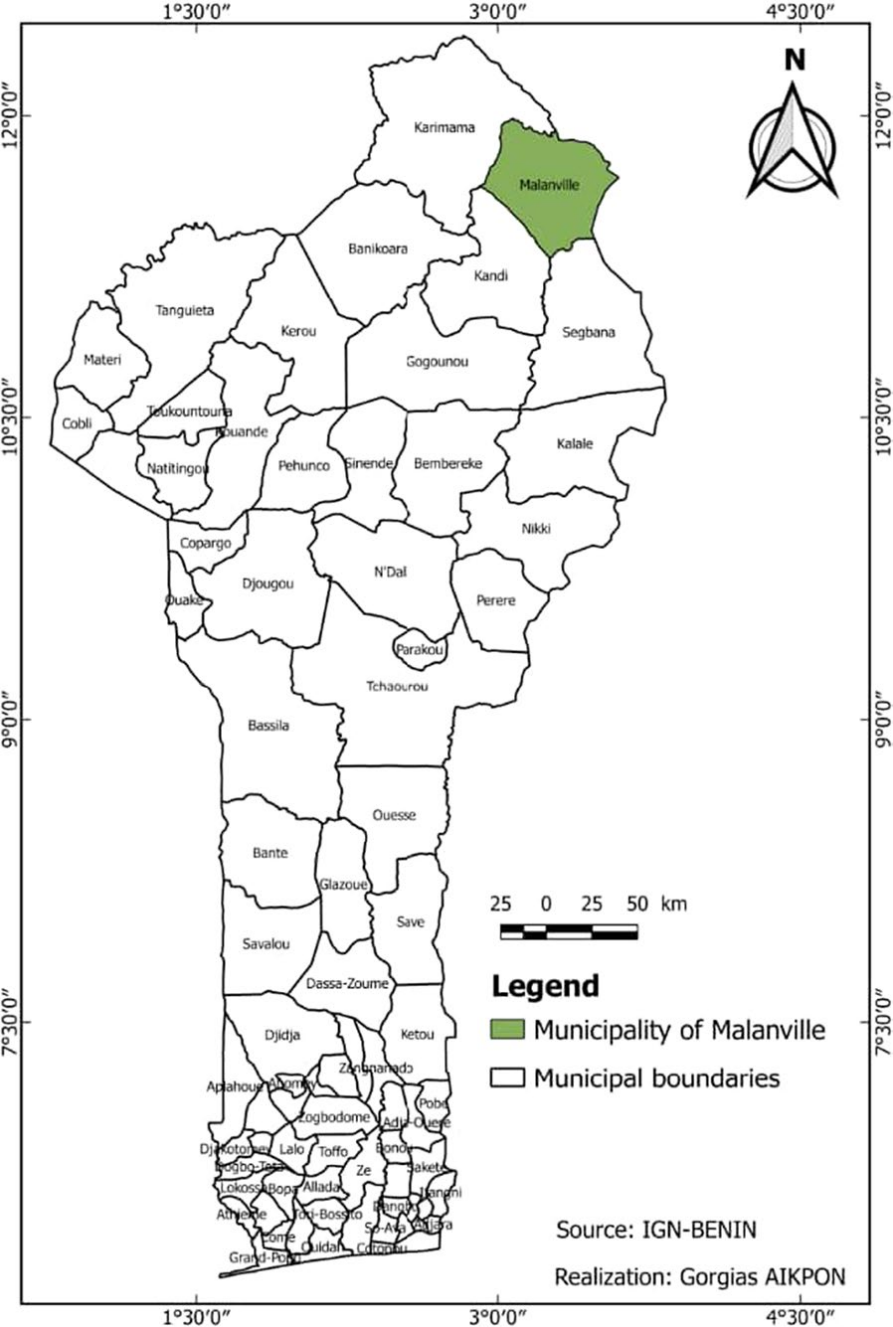
Map of Benin showing the study site

The study was conducted from March, 2016 to April, 2017 in the Malanville district (11°52 N–3°23 E) located in northern Benin (Fig. 1). The district is a rice-growing area that is surrounded by rice farms, particularly towards the North on the right bank of the Niger. The farms are settled on 516 hectares of land that spans through the entire Malanville district that is also a home for more than a million people. Farmers in this farm settlement use the continuous flooding with irrigation water pumped directly from the river Niger. Rice cultivation is done throughout the year, which suggests high malaria incidence all year round, due to the continuous flooding system of their rice field [25].

### Retrospective clinical malaria data collection

To assess the impact of irrigation technologies on malaria risk, monthly records of confirmed malaria cases were collected from Malanville Health Centre for 3 years (2013, 2014 and 2015) before this study. The records were sorted out by districts and type of malaria parasite, as confirmed by microscopy. Monthly malaria incidence was calculated for each village to determine their level of malaria transmission in all the months of the year.

### Knowledge-attitude-practices (KAP) survey among rice farmers

This survey was conducted between February and March, 2016. Prior to the commencement of the study, households were randomly selected for inclusion in the study from rosters provided by the president of rice producers. Voluntary individual farmers (household head) who gave their consent to the study were interviewed using a well-structured questionnaire [26]. Interviews were conducted in private to reduce bias from other members of the community. The languages used for the interviews were French, Hausa and Germa. The questionnaire was administered to know: (i) if local farmers have baseline knowledge on the causes of malaria, (ii) if they know the clinical symptoms of malaria, (iii) if they have any knowledge about mosquito breeding sites and mosquito larvae and (iv) the periods of mosquito abundance in their community.

### Investigations on land use, water management and mosquito breeding site identification

The survey commenced after an ethical clearance (N°19-21/12/2015) was obtained from the Ministry of Health, Benin Republic via le Comité d’Ethique pour la Recherche en Santé of Centre de Recherche Entomologique de Cotonou (CERS-CREC). Also, there was a formal agreement with rice farmers to carry out the study after the aim and objectives of the research have been fully explained and understood. In collaboration with the president of the Rice Farmers’ Association (RFA), the full list of farmers in the communities was received. From this list, 32 rice farmers as participants were strategically and randomly selected using some selection criteria, such as farm size (20 rice plots and above), age (20–50 years), gender (male or female).

Information related to soil preparation, tillage equipment and depth (deep, minimal or without tillage on rice field), land levelling after tillage (Normal or abnormal) and water management (types of irrigation system, irrigation scheme, flooding period of rice plots), presence of mosquitoes breeding sites, *Anopheles* larvae were recorded through direct observation (DO) during periods when farming activities are ongoing. Informations obtained from the DO were further confirmed during focus group discussions with the farmers.

### Experimental design

To access the impact of the new agricultural technologies (minimal tillage and intermittent flooding) on the reduction of *Anopheles* breeding sites in rice fields, an experimental field of 250 m^2^ area in the rice field was set aside for the study. On the experimental rice field, rice bins of dimensions, length: 5.5 m; width: 3.0 m; area: 16.5 m2 was set together with other factors to assess continuous flooding (CF) or intermittent flooding (IF), deep tillage (DT) or minimal tillage (MT) and normal levelling (NL) or abnormal levelling (AL). The minimal tillage means the tillage depth is less than 15 cm [12]. For intermittent flooding test plots, water was always released into plots for 7 days and left for 2 days before being irrigated again. To be sure that this process is followed duly, rice plots racks are opened and closed by farmers under the supervision of a field technician (Additional file 1: Fig. S1). The use of agrochemicals (herbicides, pesticides and insecticides) was avoided by all farmers in both the test and control plots.

Test and control plots were selected alongside with the farm owners and comparison of data obtained was done. In the control plots, farmers used common traditional practices (deep tillage, continuous watering/flooding) while in the test plots a new technology known as minimal tillage and intermittent flooding of plots was used. Five treatments including three test bins and two control bins were done for this comparison. The five treatments were set up with three (3) replicates as shown in Additional file 1: Fig. S2.

- Control bin A1, A2, A3: Plots with conventional system (Deep tillage + Continuous flooding + Abnormal levelling) DT + CF + AL.
- Test bin B1, B2, B3: Plots with one of the new technologies introduced (Minimal tillage + Continuous flooding + Abnormal levelling) MT + CF + AL.
- Test bin C1, C2, C3: Plots with one of the new technologies introduced (Deep tillage + Intermittent flooding + Abnormal levelling) DT + IF + AL.
- Control bin D1, D2, D3: Plots with conventional system (Deep tillage + Continuous flooding + Normal levelling) DT + CF + NL.
- Test bin E1, E2, E3: Plots with the two new technology introduced (Minimal tillage + Intermittent flooding + Abnormal levelling) MT + IF + AL.

### Evaluation of the performance of irrigation technologies on *Anopheles* larval development in rice farming

The contribution of minimal tillage and intermittent flooding to the reduction of mosquito larval breeding sites in rice fields was assessed by monitoring mosquito breeding sites. Control and test plots during each developmental stage of rice (transplanting, tillering and maturation) were checked for the presence of water pockets, which could serve as mosquito breeding site by small group of farmers accompanied by an entomologist. Direct observations of mosquito breeding sites, presence of *Anopheles* mosquito larval, breeding site size, larval development, larval densities and larval persistence were all assessed.

Mosquito larvae were collected from 10 am to 2 pm in the test and control bins during the different stages of rice development using the dipping method [27]. In each plot, 20 scoops were taken using a standard white 350 ml dipper (ladle). Mosquito larvae collected were identified morphologically using the Gillies and Meillon identification keys and technically recorded based on the collection points [28].

### Data analysis

The total number of mosquito and *Anopheles* larvae collected per year per bin and rice development stages was recorded in Excel. The significant difference between the type of irrigation system and the density of mosquito larvae was analyzed with ANOVA using XLSTAT software, 2011.

## Results

### Knowledge and prevention strategy of malaria by rice farmers from Malanville

The farmers’ knowledge and practices about malaria, including its transmission and preventive measures is presented in Table 1. A total number of 104 households heads were interviewed with 29% as females while 71% were males. Malaria (89.42%) and schistosomiasis (27.88%) were the main diseases observed in Malanville, with over half of the participants, 54.80% having being earlier sensitized about malaria disease.

**Table 1:** Farmer’s knowledge on malaria

Variables	Number	Percentage	P
Awareness about malaria			
Yes	57	54.80%	0.1
No	47	45.20%	
Diseases suffering by rice farmers			
Malaria	93	89.42%	0.001
Yellow fever	5	4.80%	
Schistosomiases	29	27.88%	
Athlete foot	11	10.57%	
Causes of Malaria			
Mosquito bites	89	85.57%	0.001
Hardwork	16	15.38%	
Sun	20	19.23%	
Dirty water consumption	41	39.42%	
Oil consumption	15	14.42%	
Witchcraft	3	2.88%	
Sign of malaria			
Fever	83	79.80%	0.001
Headache	18	17.30%	
Vomiting	61	58.65%	
Diarrhoea	22	21.15%	
Other	30	28.84%	
Mosquitoes breeding areas			
Dirty stagnant water	93	89.42%	0.001
Bushes	38	36.53%	
Rice field	5	4.80%	
Others	3	2.88%	
Mosquito larvae known			
Yes	9	8.65%	0.00001
No	95	91.34%	
Period of mosquito abundance			
Rainy season	68	65.38%	0.01
Dry season	9	8.65%	
During rice cultivation	59	56.73%	
Don’t know	1	0.96%	

The majority (85.57%) of respondents have adequate understanding that mosquito transmits malaria through bite, although with few misconceptions that are expected. Some believed that drinking dirty water (39.42%), exposure to the sun (19.23%), working hard at all times (15.38%) or oil consumption (14.42%) could also cause malaria. Stagnant water as a main breeding site of mosquitoes was mentioned by 89.42% of respondents. However, 5 farmers out of 104 (4.80%) have a prior knowledge that rice growing areas could be a potential mosquito breeding site. Symptoms of malaria such as fever, headache, vomiting and diarrhea were frequently mentioned by farmers. Most of the respondents (91.34%) did not recognize mosquito larvae. Also, a large number of participants (91.34%) knew that rainy season and rice cultivation period are proper time for mosquito proliferation.

Strategies used for malaria prevention were the use of insecticidal bed nets (94.23%), clean the vegetation surrounding the house (85.57%), mosquito coil (75%) and insecticide aerosol sprays (17.30%). About 84.61% of households declared that they received bed nets from the free distribution campaign organized by the National malaria control program of the Ministry of Health whereas 28.84% of respondents purchased theirs from local markets.

### Impact of irrigation practices in rice field on malaria transmission

**Fig. 2:**
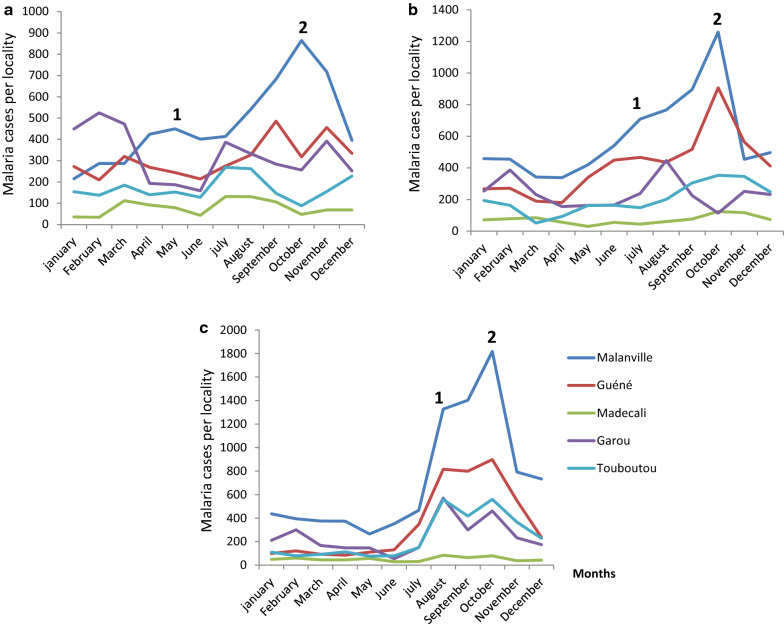
Malaria cases in Malanville district in 2013 (**a**), 2014 (**b**) and in 2015 (**c**). NB: This figure reveals two peaks of transmission: peak 1 in July due to the rainy season and peak 2 in September due to the rice production and rainfall

Farmers understand that there is always a rapid increase of mosquito’s population during the rice production periods. They were also able to associate the high number of malaria cases occurred in the households during the growing seasons with the rapid increase of mosquito density. Malaria epidemiological data from 2013 to 2015 in Malanville district obtained from the health authority confirmed the farmer’s observations (Fig. 2).

The data showed that malaria cases increased during the rainy season and the dry season when intensive rice production is ongoing. The number of malaria cases was highest in the urban centre of Malanville with two peaks. The first peak was in July in all localities and corresponded to the rainy season, while the second one appeared in October and corresponded to the rice irrigation period.

In Malanville centre, malaria cases were highest in October/November, which often coincides with intensive rice production (Fig. 2).

### Impact of minimal tillage and intermittent flooding on mosquito larval densities

**Fig. 3:**
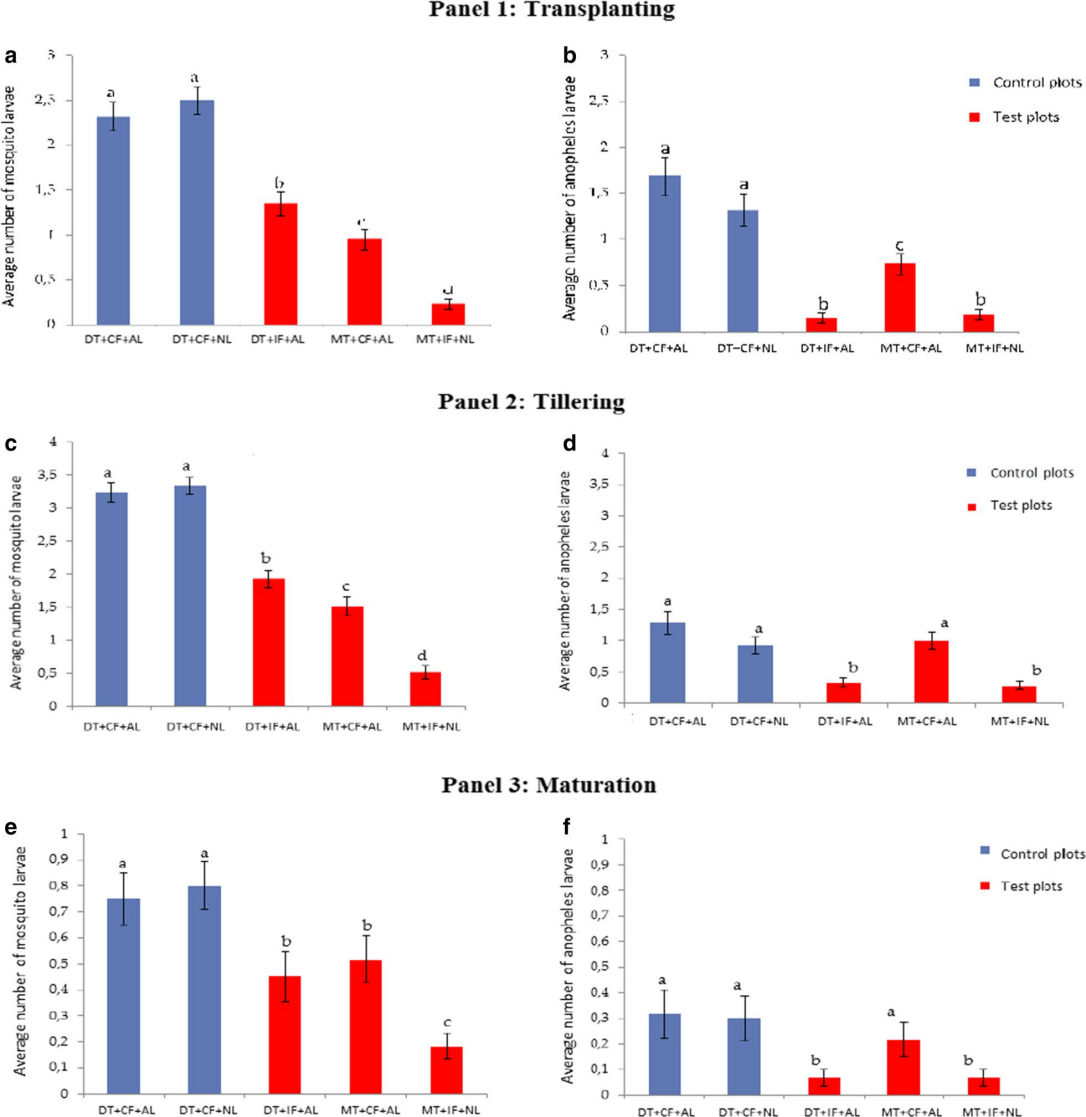
Mean density of all mosquito larvae and *Anopheles* mosquito larvae collected during Panel 1 transplanting (**a**, **b**), Panel 2 tellering (**c**, **d**) and Panel 3 maturation (**e**, **f**). NB: DT, Deep tillage; MT, minimal tillage; CF, continuous flooding; IF, intermittent flooding; NL, normal levelling; AL, abnormal levelling. *Histogram bars sharing the same letters are not significantly different*

During the three developmental stages of rice (transplanting, tillering and maturation) mosquito larvae were collected and the mean densities were compared between tillage and irrigation system. Larval density varied during the three developmental stages of rice; during the transplanting stage, the density of mosquito larvae especially *Anopheles* larvae was significantly reduced (P = 0.0000) with minimal tillage, intermittent flooding and normal leveling (MT + IF + NL) compared to the combination of deep tillage, continuous flooding and abnormal levelling (DT + CF + AL). The MT + IF + NL and DT + IF + NL significantly reduced the density of anopheles larvae compared to MT + CF + AL, which was more effective larvae reduction compared to DT + CF + NL and DT + CF + AL (Fig. 3). Overall, the MT + IF + NL combination was observed to reduce *Anopheles* larvae density by 80.83 times (P = 0.0000).

During the tillering stage, MT + IF + PN and DT + IF + AL significantly reduced larval density than MT + CF + AL, DT + CF + NL and DT + CF + AL with MT + IF + PN combination reducing anopheles larva density by 30.88 times during the tillering stage. Similar results were obtained during the maturation stages of rice. The combination of MT + IF + PN reduced larval density by 40.75 times during the maturation (Fig. 3). However, the pattern of levelling did not have any effect (P > 0.05) on the reduction of mosquito larvae density during all the developmental stages (Table 2).

**Table 2:** Mosquito larvae density/350 ml according to different technologies during rice development

	Tillage		Flooding		Leveling	
	Deep	Minimal	Continuous	Intermittent	Normal	Abnormal
Transplanting	2,056^a^	0,592^b^	1,922^a^	0,792^b^	1,367^a^	1,539^a^
P value	0,000000		0,000000		0,088302	
Tillering	2,833^a^	1,017^b^	2,694^a^	1,225^b^	1,925^a^	2,228^a^
P value	0,000000		0,000000		0,151127	
Maturation	0,667^a^	0,350^b^	0,689^a^	0,317^b^	0,492^a^	0,572^a^
P value	0,001351		0,000065		0,818796	

NB, The numbers sharing the same letter are not significantly different. Comparison was made between deep tillage and minimal tillage, continuous flooding and intermittent flooding, normal levelling and abnormal levelling effect on reduction of anopheles larval density

## Discussion

Irrigated agriculture is necessary for the expansion of agricultural productivity and to ensure food security. However, irrigation systems have been suggested as one of the contributing factors to increase malaria incidence in many parts of sub-Saharan Africa. The availability of water pockets for mosquito breeding during irrigation appeared to spread the malaria disease and contribute to the extension of disease transmission into the dry season in agricultural areas. However, proper land use and water management, by improving irrigation technologies could help to mitigate this challenge. In the present study, a field experiment was carried out to assess the impact of land use and water management practices on *Anopheles* larval density in the rice field at Malanville.

The combination of minimal tillage system with intermittent flooding has a great potential to reduce anopheles breeding sites and larval density in rice fields, as previously observed in other regions in Africa [29–31]. A study conducted in western Ethiopia reported that higher malaria prevalence and transmission risk increased due to high vector abundance in the irrigated sugarcane agroecosystem than non-irrigated agroecosystem [30]. Mutero et al. [32] observed that the use of intermittent flooding in Mwea rice irrigation scheme in Kenya yielded a lower mosquito larval densities and survival. Similarly, Kibret et al. [33] noticed a reduced density of *Anopheles* in villages where intermittent flooding was used for irrigation in Ethiopia. In China, Qunhua et al. [34] also reported a high reduction of vector breeding below the level required to sustain malaria transmission among individuals in rice field’s areas. Furthermore, in 4-year large-scale experiments conducted in Portugal, it was suggested that intermittent flooding of rice fields does not change the quantity or quality of rice yields. On the contrary, increase in yield was reported in some studies, together with a reduction of over 80% in the number of anopheline larvae [35].

The excessive proliferation of anopheles mosquito vectors due to the type of irrigation system will undoubtedly lead to a higher risk of malaria transmission [31]. Similar concerns were observed from literature where studies have reported a negative impact of some certain practices in Agriculture on the risk of public health diseases [36, 37]. Continuous flooding of rice fields during irrigation was implicated for creating more breeding sites for *An. gambiae* than normal, which ensured a continual transmission of malaria [36]. A re-orientation and continuous education about this situation are needed so that farmers can know how their activities on the farm can influence and increase the risk of diseases transmission such as malaria and how this will continue if their attitudes towards these are not changed.

Data of malaria cases collected from the health centre at Malanville showed a link between the rice production and the malaria incidence. In Malanville urban centre, locality surrounded by rice farms, the second peak of malaria transmission is not only linked to the end of the rainy season but also and especially to the production of rice. On the other hand, in Guéné, Garou, Madecali and Touboutou, where there is no rice production, the peak of the disease is only recorded at the end of the rainy season with the availability of water pockets favouring the reproduction of *Anopheles*.

These observations make continuous farmer’s engagement and sensitization on the recognition of various environmental factors involved in the proliferation of mosquitoes in rural communities very important. The type of irrigation systems used in rice fields has been shown to influence increasing abundance of main malaria vector species, *An. gambiae* sensu lato (*s.l.*) and *An. funestus s.l.* [4, 7, 16, 38] and others potential vectors, such as *Anopheles pharoensis* [4, 33, 39–41], *Anopheles rufipes* [15, 42, 43], *Anopheles ziemanni*, frequently found in the irrigation canals of the paddies field [7]. Also, there has been observations of a significant increase of *Anopheles* densities in irrigated rice field compared to areas with non-irrigated system [8, 44]. Besides, its contribution to malaria vector abundance has impacted high *Anopheles* species occurrence, distribution and diversity [16]. Indeed, all stages of rice cultivation (land preparation, nursery preparation, transplanting, fertilizer application, field maintenance, pre-harvesting drainage, and harvesting) were observed to be contributing to this spread.

This cropping cycle also impacted temporal distribution and abundance of malaria vectors. The observed association between continuous flooding and high larval vectors densities compared to intermittent flooding suggests that intermittent flooding should be considered over continuous flooding during rice cultivation. This is not only for the reduction of malaria risks among individuals by minimizing unwanted waterlog on/around the farm and halting mosquito larvae developmental stages but also for water management strategy.

Further research is however, necessary to, among other things; determine whether rice yields could be increased by using intermittent flooding in Malanville. It would likewise be important to assess on a wider scale the feasibility of implementing intermittent flooding with respect to farmer acceptance and required changes in irrigation system design and management.

## Conclusion

This study revealed that continuous flooding systems in rice field influences higher proliferation of malaria vectors and consequentially increases the risk of malaria transmission in Malanville district. Instead, an improved irrigation strategy, intermittent flooding, which does not only help to manage water usage but also has the capacity to reduce mosquito breeding sites hence reduces malaria transmission is suggested for use in rice cultivation. While training farmers on how to use intermittent flooding and showing how it will help reduce malaria transmission, it would also be necessary to establish that this alternative method would increase rice yield. This will engender quick and more acceptance of the method, hence, further investigation on rice yield rate of this method at Malanville. This information can be disseminated through organised education programmes for farmers to assist them to check mate all agricultural practices. This will help to prevent unwanted health cases as a result of poor decisions made on the farm in order to reduce larval and adult vector abundance and hence lower the risk of malaria transmission.

## Supplementary information

**Additional file 1: Figure S1.** a) water supply, b) water released from rice plots. **Figure S2.** Experimental set of rice plots made in the Malanville rice area. **Figure S3.** Equipment and tillage system in the rice-growing area of Malanville. a: Hoe tillage, b: Plow tillage, c: tillage using tiller. **Figure S4.** Irrigation systems relying on water storage (a); the channel (b) and the pumping system (c).

## Data Availability

All data generated and analysed during this study are included in the manuscript and its Additional file 1.

## Abbreviations

DT: Deep tillage
MT: Minimal tillage
CF: Continuous flooding
IF: Intermittent flooding
NL: Normal levelling
AL: Abnormal levelling
