# Supplementary material for: Minimal tillage and intermittent flooding farming systems show a potential reduction in the proliferation of Anopheles mosquito larvae in a rice field in Malanville, Northern Benin
Source: Malar J. 2020 Sep 14;19:333. doi: 10.1186/s12936-020-03406-2 (PMC7491134; doi:10.1186/s12936-020-03406-2)
Supplement: Supplementary file 1 — Additional file 1: Figure S1. a) water supply, b) water released from rice plots. Figure S2. Experimental set of rice plots made in the Malanville rice area. Figure S3. Equipment and tillage system in the rice-growing area of Malanville. a: Hoe tillage, b: Plow tillage, c: tillage using tiller. Figure S4. Irrigation systems relying on water storage (a); the channel (b) and the pumping system (c). [file 12936_2020_3406_MOESM1_ESM.docx]

**Additional file 1**

**b**

**a**

**Figure S1:** a) water supply, b) water released from rice plots


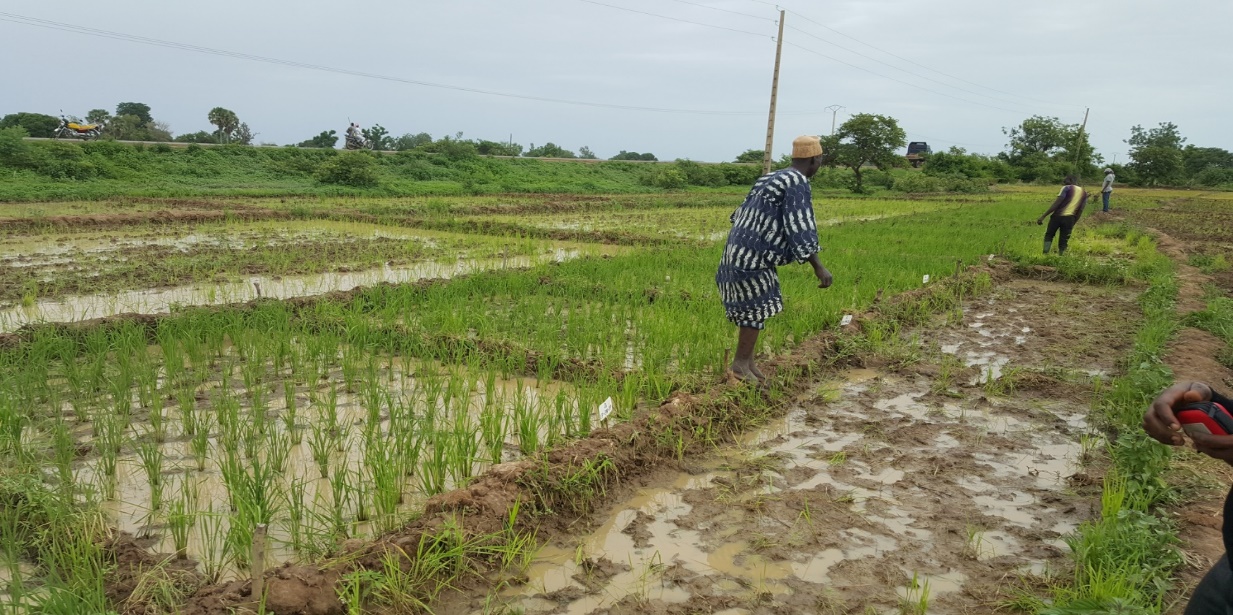


**Figure S2:** Experimental set of rice plots made in the Malanville rice area


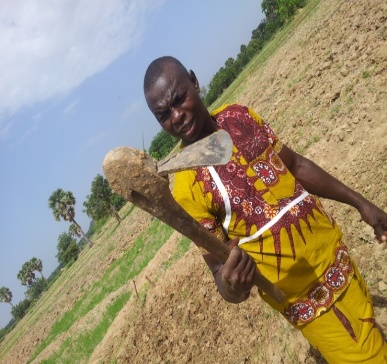

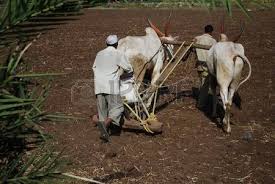

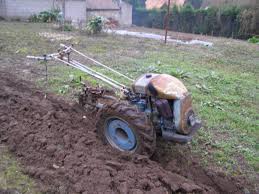


**c**

**b**

**a**

**Figure S3**: Equipment and tillage system in the rice-growing area of Malanville
a: Hoe tillage, b: Plow tillage, c: tillage using tiller.


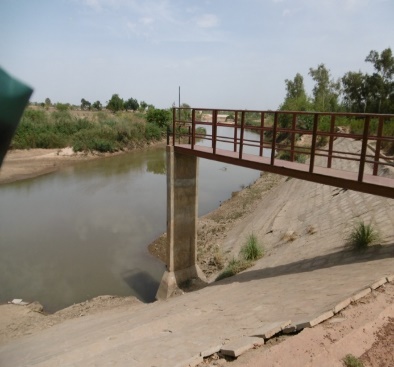

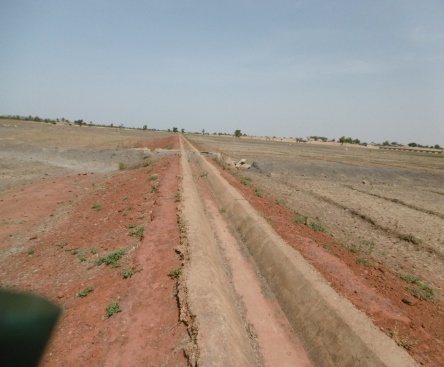

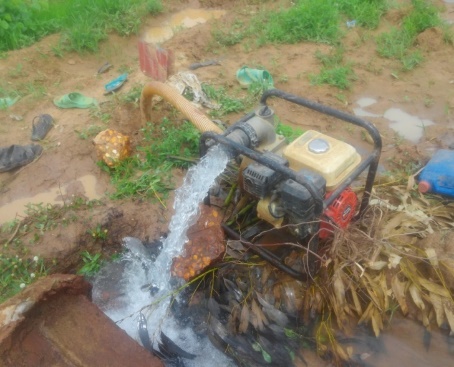


**c**

**b**

**a**

**Figure S4:** Irrigation systems relying on water storage (a); the channel (b) and the pumping system (c)
